# Effects of Traditional and Innovative Safe Breastfeeding Pillows on Postpartum Maternal Breastfeeding Self‐Efficacy, Awareness, and Comfort: A Randomized Controlled Trial

**DOI:** 10.1155/jonm/8414086

**Published:** 2026-06-16

**Authors:** Ju-Fen Chou, Chu-Hsuan Kuo, Ka-Wai Tam, Hsiu-Ju Jen, Wen-Pei Chang, Yun-Yun Chou, Kee-Hsin Chen

**Affiliations:** ^1^ Department of Nursing, Shuang Ho Hospital, Taipei Medical University, New Taipei City, Taiwan, tmu.edu.tw; ^2^ Department of Obstetrics & Gynecology, St. John’s Episcopal Hospital, Far Rockaway, New York, USA, ehs.org; ^3^ Division of General Surgery, Department of Surgery, Shuang Ho Hospital, Taipei Medical University, New Taipei City, Taiwan, tmu.edu.tw; ^4^ Division of General Surgery, Department of Surgery, School of Medicine, College of Medicine, Taipei Medical University, Taipei, Taiwan, tmu.edu.tw; ^5^ Cochrane Taiwan, Taipei Medical University, Taipei, Taiwan, tmu.edu.tw; ^6^ School of Nursing, College of Nursing, Taipei Medical University, Taipei, Taiwan, tmu.edu.tw; ^7^ Shared Decision Making Resource Center, Shuang Ho Hospital, Taipei Medical University, New Taipei City, Taiwan, tmu.edu.tw; ^8^ Post-Baccalaureate Program in Nursing, College of Nursing, Taipei Medical University, Taipei, Taiwan, tmu.edu.tw; ^9^ Department of Nursing and Research Center in Nursing Clinical Practice, Wan Fang Hospital, Taipei Medical University, Taipei, Taiwan, tmu.edu.tw; ^10^ Evidence-based Knowledge Translation Center, Wan Fang Hospital, Taipei Medical University, Taipei, Taiwan, tmu.edu.tw; ^11^ School of Medicine, Faculty of Health and Medical Sciences, Taylor’s University, Subang Jaya, Malaysia, taylors.edu.my

**Keywords:** breastfeeding, innovative breastfeeding pillow, maternal comfort, postpartum mother, randomized controlled trial

## Abstract

**Objective:**

To evaluate the effectiveness of an innovative “Safe Breastfeeding Pillow” on postpartum maternal self‐efficacy, awareness, and comfort during breastfeeding.

**Methods:**

A participant‐ and outcome assessor–blinded, parallel randomized controlled trial was conducted. Postpartum mothers were randomized into intervention and control groups. The intervention group used the innovative Safe Breastfeeding Pillow, which includes a main pillow and an additional pillow that can be wrapped around the baby to maintain a stable feeding posture and reduce the risk of accidents due to maternal fatigue during breastfeeding. The control group used a U‐shaped breastfeeding pillow as usual. Outcome measures included the Breastfeeding Self‐Efficacy Scale–Short Form (BSES‐SF), Breastfeeding Awareness Scale (BAS), Body Part Discomfort Scale (BPDS), and B‐R‐E‐A‐S‐T‐Feed Observation Form. Statistical analyses included Chi‐squared tests, independent *t-*tests, and linear regression.

**Results:**

In total, 128 mothers (64 each in the intervention and control groups) were enrolled. No differences were found between the two groups in demographic characteristics. The BSES‐SF did not significantly differ between the groups. The intervention group reported significantly higher scores for the BAS (3.58 ± 0.04 vs. 1.60 ± 0.15, *p* < 0.0001). In the BPDS, the intervention group showed significantly higher comfort levels in all body parts during breastfeeding compared to the control group (3.52 ± 0.13 vs. 1.70 ± 0.33, *p* < 0.0001). Both groups exhibited increased emotional bonding at the initial assessment and at discharge according to the B‐R‐E‐A‐S‐T‐Feed Observation Form.

**Conclusions:**

The innovative Safe Breastfeeding Pillow effectively improved maternal comfort, safety, and breastfeeding awareness compared to a traditional pillow. It provides essential support and warmth, enhancing the overall breastfeeding experience and maternal well‐being.

**Implications for Nursing Management:**

Nursing leaders should incorporate the “Safe Breastfeeding Pillow” into routine care as a key strategy to ensure feeding safety, enhance maternal comfort, and improve breastfeeding rates.

**Trial Registration:** ClinicalTrials.gov_identifier: NCT05687383

**Summary**
• What is already known
◦ Breastfeeding is crucial for infant health; yet challenges such as discomfort and safety concerns often hinder its practice.
◦ A traditional U‐shaped breastfeeding pillow is commonly placed on the mother’s thigh to provide a comfortable platform for placing the baby.
• What this paper adds
◦ The nurse‐designed innovative Safe Breastfeeding Pillow can be wrapped around the baby to maintain a stable feeding posture and reduce the risk of accidents due to maternal fatigue during breastfeeding.
◦ The innovative Safe Breastfeeding Pillow effectively improved maternal awareness of breastfeeding safety, comfort, and emotional bounding compared to traditional pillows.
◦ Nursing leaders should incorporate the “Safe Breastfeeding Pillow” into routine care as a key strategy to ensure feeding safety, enhance maternal comfort, and improve breastfeeding rates.

## 1. Introduction

Breastfeeding provides personalized nutrition for infants and health benefits for mothers, but it is not as widely practiced as it should be. Less than half of newborns begin breastfeeding within an hour of birth, and just 44% of infants under 6 months are exclusively breastfed worldwide [1, 2]. In the postpartum period, both women and newborns are vulnerable. Prolonged periods of breastfeeding result in maternal exhaustion and muscle strain due to incorrect positioning, discomfort, and potential increased risks of accidental incidents with the baby, which in turn may diminish the mother’s motivation to continue breastfeeding [3–5].

The traditional U‐shaped breastfeeding pillow, placed on the mother’s thigh, provides a comfortable platform to place the baby. Several studies confirmed its effectiveness in alleviating maternal discomfort during breastfeeding [6, 7]. Nevertheless, continuous hand support from the mother is essential to maintain the correct position and prevent accidents, particularly during nighttime feedings when there is a risk of unintentional incidents if the mother falls asleep. Many mothers (approximately 70.3%) discontinue breastfeeding due to challenges with sustaining a comfortable and proper breastfeeding posture, resulting in issues such as cracked nipples, an insufficient milk supply, pain, and exhaustion [8, 9]. Remarkably, our hospital’s exclusive breastfeeding rate in 2022 averaged only 9.83%, marking significant declines from 28.57% in 2019 and 16.48% in 2020.

Because breastfeeding is frequent and each feeding session takes an amount of time during the early neonatal period, many mothers experience fatigue, musculoskeletal discomfort, and concerns about infant safety during breastfeeding. We hypothesize that the absence of a more effective, secure, and hands‐free breastfeeding support tool may contribute to suboptimal breastfeeding outcomes, underscoring the need for the development of an innovative breastfeeding pillow. For nurse leaders, this issue is particularly important because they play a critical role in shaping breastfeeding‐supportive care environments, guiding evidence‐based postpartum practices, and introducing innovations that improve maternal comfort, confidence, and breastfeeding success. Their influence extends beyond direct bedside nursing care to staff education, guideline development, resource allocation, and the creation of practice infrastructures that support breastfeeding initiation and continuation. In this context, supportive devices that effectively address common breastfeeding challenges, including poor positioning, physical strain, and discomfort, may represent a meaningful care innovation that enhances maternal comfort, supports breastfeeding success, and strengthens the quality of postpartum maternal–newborn care.

**FIGURE 1:**
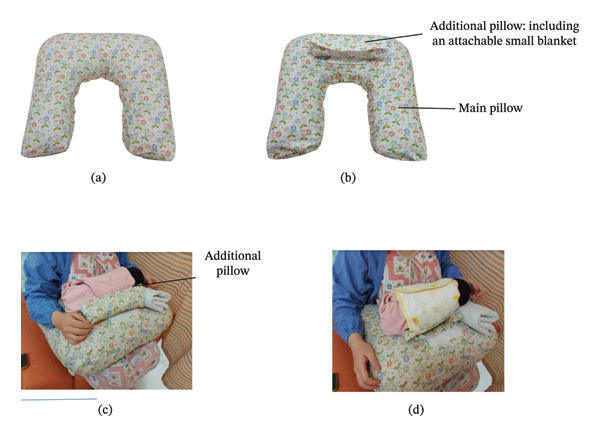
Traditional breastfeeding pillow and innovation “Safe Breastfeeding Pillow.” (a) Traditional breastfeeding pillow. The traditional U‐shaped breastfeeding pillow allows support when breastfeeding the baby. (b) The innovation safe breastfeeding pillow includes a main pillow and an additional pillow. (c) Innovative safe breastfeeding pillow usage. The additional pillow supports the infant’s back, ensuring comfortable and secure breastfeeding. It connects to the main pillow and can be adjusted to fit the infant’s size, reducing the risk of accidents due to maternal fatigue. (d) Function of the additional pillow. The additional pillow includes a small blanket that can be wrapped around the baby to maintain a stable feeding posture and provide warmth during nighttime feedings or when the baby’s temperature is low.

To improve upon the traditional breastfeeding pillow, we designed an innovative “Safe Breastfeeding Pillow” which incorporates a detachable small blanket secured with Velcro which can be converted into a pillow (Figure 1). The Safe Breastfeeding Pillow was designed based on the dimensions and weight of traditional U‐shaped breastfeeding pillows, measuring 56 × 62 × 8 cm and weighing 2150 g. The Safe Breastfeeding Pillow includes a detachable blanket available in sizes appropriate for both full‐term and preterm infants. For preterm infants, a smaller S‐size pillow is provided to ensure optimal positioning, support, and safety during breastfeeding. It mimics the mother’s hands by wrapping around the baby to help the baby maintain a secure feeding position, ensuring safety and comfort during nursing, while also preventing the mother from becoming tired. It can also serve as a warm blanket during nighttime feedings or when the baby’s temperature is lower than desired. Its use extends beyond hospital settings, offering benefits in community and home environments by supporting maternal well‐being. With innovative improvements, it is user‐friendly and requires no specialized training while promoting increased breastfeeding safety and medical satisfaction and elevating the quality of care for mothers and infants.

The objective of this randomized controlled trial (RCT) was to evaluate the effectiveness of the innovative Safe Breastfeeding Pillow in improving postpartum maternal breastfeeding self‐efficacy, awareness, and comfort compared with a traditional breastfeeding pillow. We hypothesized that mothers using the Safe Breastfeeding Pillow would demonstrate higher levels of breastfeeding self‐efficacy, greater breastfeeding awareness, and improved breastfeeding comfort than those using a traditional breastfeeding pillow.

## 2. Methods

### 2.1. Study Design and Ethical Considerations

This was a participant‐ and outcome assessor–blinded, parallel, randomized controlled study. The study protocol was approved by the Joint Institutional Review Board of Taipei Medical University (approval no.: N202210042). Before data were collected, all participants were informed of the study aims and procedures and signed an informed consent form.

### 2.2. Setting and Participants

Participants were recruited from the same postpartum unit at Shuang Ho Hospital, Taipei Medical University (New Taipei City, Taiwan), from February to December 2023. Inclusion criteria included postpartum mothers who voluntarily chose to participate in the study, experienced a vaginal delivery without severe postpartum complications (e.g., postpartum hemorrhage, preeclampsia, and severe perineal trauma), and had an infant capable of adapting to the cradle position. Additionally, participants were required to engage in breastfeeding their infants at least three times a day, with each feeding session lasting a minimum of 10 min. Exclusion criteria applied to (1) infants whose health status changed after birth; (2) mothers with significant physical illnesses that hindered continuous breastfeeding (e.g., severe anemia, uncontrolled hypertension, and postpartum complications); (3) those unable to communicate in Mandarin or Taiwanese; and (4) patients who failed to respond to all questions.

#### 2.2.1. Sample Size Estimation

Sample size estimation was performed using G∗Power 3.1. An a priori power analysis was conducted for a two‐tailed independent‐samples *t*‐test comparing two groups. Drawing on the sample size estimation approach reported in a previous breastfeeding pillow trial [10], the analysis was based on a medium effect size (*d* = 0.50), a significance level of 0.05, a statistical power of 0.80, and a 1:1 allocation ratio. The analysis indicated that a total of 128 participants would be required, with 64 participants per group.

### 2.3. Randomization and Blinding

After delivery, postpartum mothers were randomly allocated to either an intervention group, that received the innovative Safe Breastfeeding Pillow, or a control group, supplied with a traditional breastfeeding pillow, all done through computer software. The allocation sequence was concealed in sequentially numbered opaque, sealed envelopes from the principal investigator who enrolled and evaluated participants. Both groups received a breastfeeding pillow, and participants were blinded to the type of pillow they received. The healthcare providers responsible for distributing the breastfeeding pillows were not part of the blinding process. Subsequently, data collectors, uninvolved in the intervention, collected questionnaire data in the ward after mothers had completed their feeding, and they were also blinded to the type of pillow used by the participants.

### 2.4. Intervention Process

**FIGURE 2:**
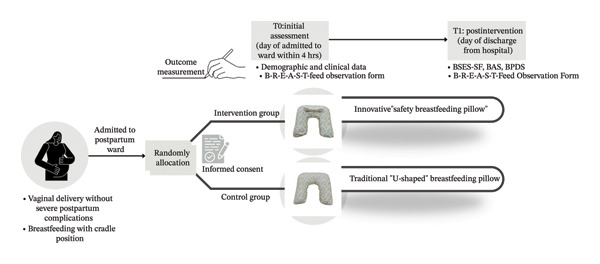
Flowchart of the study process. Note: BSES‐SF, Breastfeeding Self‐Efficacy Scale–Short Form; BAS, Breastfeeding Awareness Scale; BPDS, Body Part Discomfort Scale.

After delivery, mothers were admitted to the postpartum ward and began to breastfeed their infants. Nursing staff provided breastfeeding education and actively assisted mothers and infants in adopting appropriate breastfeeding positions within 4 h of admission to the ward. In general, nursing staff will place a breastfeeding pillow on a mother’s thighs in the cradle breastfeeding position to help support the positioning of both the mother and infant. In this study, both groups received a breastfeeding pillow: The control group received a traditional U‐shaped breastfeeding pillow, and the intervention group was supplied with the innovative Safe Breastfeeding Pillow. Apart from the different types of breastfeeding pillow, both groups received identical clinical assessments and care procedures. Research team members collected basic information at the first breastfeeding session. Postpartum mothers from both groups independently completed the questionnaires of basic information, the Breastfeeding Self–Efficacy Scale‐Short Form (BSES‐SF), Breastfeeding Awareness Scale (BAS), and The Body Part Discomfort Scale (BPDS) on the day of discharge from the hospital. The B‐R‐E‐A‐S‐T‐Feed Observation Form was recorded by nursing staff in the nursery room during the first breastfeeding session (within 4 h of being admitted to the postpartum ward) and last breastfeeding session (on the day of discharge from the hospital). Clinical data of the mother and newborn were obtained from medical records. Prior to the study, nursing staff underwent standardized education and training of innovative Safe Breastfeeding Pillow and traditional U‐shaped breastfeeding pillow according to the ward’s standard care procedure to ensure the homogeneity of the educational intervention. A flowchart of the study process is shown in Figure 2.

#### 2.4.1. Control Group: Traditional U‐Shaped Breastfeeding Pillow (Usual Care)

In the control group, mothers engaged in breastfeeding their infants in the cradle position at least three times a day, with each feeding session lasting a minimum of 10 min. Nursing staff aided the mothers by positioning the U‐shaped breastfeeding pillow on the mother’s thigh to provide a comfortable platform for placing the baby, enabling an ideal breastfeeding position with the baby facing the mother (Figure 1A).

#### 2.4.2. Intervention Group: Innovative Safe Breastfeeding Pillow

In the intervention group, mothers also engaged in breastfeeding their infants in the cradle position at least three times a day, with each feeding session lasting a minimum of 10 min. In the innovation group, the Safe Breastfeeding Pillow designed by nursing staff was used. This pillow introduced an inventive feature: an attachable small blanket secured with Velcro. The entire pillow includes a main pillow and an additional pillow (Figure 1B). The additional pillow is connected to the main pillow and can be adjusted to fit the infant’s size, reducing the risk of accidents due to maternal fatigue. It supports the infant’s back, ensuring comfortable and secure breastfeeding. Additionally, the additional pillow includes a small blanket that can be wrapped around the baby to maintain a stable feeding posture and provide warmth during nighttime feedings or when the baby’s temperature is low (Figures 1C and 1D).

### 2.5. Outcome Measurements

Data were systematically collected through structured questionnaires. The questionnaires used in this study included the following.

#### 2.5.1. Basic Information

The basic information questionnaire included maternal age, parity status, educational level, marital status, occupation, previous breastfeeding experience, experience participating in breastfeeding workshops, infant feeding method during hospitalization, and dominant hand. For newborns, the questionnaire gathered information on gestational age, height, weight, urinary frequency, and serum bilirubin level on the day of discharge. Additional clinical data of the mother and newborn were obtained from medical records.

#### 2.5.2. Primary Outcome: The BPDS

The BPDS is used to evaluate the comfort level of different body parts of a mother during breastfeeding. There are 10 body regions in total, with a four‐point Likert‐scale ranging from 1 = *extremely uncomfortable* to 4 = *very comfortable*. The lower the points, the more uncomfortable a mother feels. The reliability of the BPDS in this study was excellent, with a Cronbach’s alpha of 0.982, indicating a high level of internal consistency.

#### 2.5.3. Secondary Outcomes

1. The BSES‐SF [11]: The BSES‐SF is a 14‐item questionnaire derived from the original 33‐item BSES. The traditional Chinese version was translated and validated by Professor Lien‐Chen Hu in 2003. The scale demonstrated good internal consistency reliability, with a Cronbach’s α of 0.93 [12]. It measures a mother’s confidence in breastfeeding, with responses ranging from 1 to 5, where 1 = *highly uncertain* and 5 = *highly confident*, and it was administered on the day of discharge. The scale evaluates various aspects of breastfeeding self‐efficacy, including maternal confidence, comfort, and ability to overcome breastfeeding challenges. A higher score reflects greater confidence and perceived ability to successfully breastfeed.
2. BAS: The BAS has four questions that use a Likert scale to evaluate the mother’s awareness and security level during breastfeeding on a scale of 1 to 4, with 1 = *extremely worried* and 4 = *very secure* for a total of 0–16 points. The higher the points, the greater awareness and safer the mother feels. The reliability of the BAS in this study was excellent, with a Cronbach’s alpha of 0.906, indicating a high level of internal consistency.
3. B‐R‐E‐A‐S‐T‐Feed Observation Form [13]: The B‐R‐E‐A‐S‐T‐Feed Observation Form, developed by the International Baby Food Action Network (IBFAN) and the United Nations International Children’s Emergency Fund (UNICEF) in 1992, is designed to assess and record various aspects during the process of breastfeeding to identify signs of both successful actions and potential difficulties. The evaluation includes six domains: body position, responses, emotional bonding, anatomy, suckling, and time spent suckling. Nursing staff recorded observations during breastfeeding sessions. In this study, each item on the B‐R‐E‐A‐S‐T‐Feed Observation Form indicating “Signs that breastfeeding is going well” was given 1 point, while items showing “Signs of possible difficulty” were assigned 0 points. For the B‐R‐E‐A‐S‐T‐Feed Observation Form, we used the traditional Chinese version provided in the breastfeeding education and training materials issued by the Health Promotion Administration, Ministry of Health and Welfare, Taiwan. As this is an officially disseminated clinical training resource, no additional translation or cultural adaptation was performed by our team.

We developed the BAS and BPDS using a hybrid approach that integrated findings from literature reviews, expert consultations, and collaborative team discussions. The questionnaires were refined through consultations with three postpartum mothers, whose feedback helped ensure relevance and clarity. Expert input was also obtained from three professionals with diverse backgrounds, including a university faculty member, a hospital nursing director, and the head nurse of a postpartum ward. The research team conducted three 2‐h meetings to design and finalize the questionnaires.

### 2.6. Statistical Analysis

Data processing and statistical analyses were conducted using SPSS for Windows, Vers. 29.0. An intention‐to‐treat analysis was employed, incorporating data from all participants. To compare demographic characteristics and measures including the BSES‐SF, BAS, BPDS, and B‐R‐E‐A‐S‐T‐Feed Observation between the intervention and control groups, Chi‐squared tests and independent‐sample *t*‐tests were used. Categorical variables are presented as counts and percentages, whereas continuous variables are reported as mean differences with SDs. Linear regression analyses were performed to examine whether the intervention effect remained significant after adjusting for clinically relevant covariates. The dependent variable in the regression model was the postintervention BPDS score. Educational level and newborn bilirubin levels were included a priori based on clinical relevance reported in breastfeeding research, in addition to study group allocation. Both crude and adjusted models were presented. Statistical significance was established at *p* < 0.05.

## 3. Results

**FIGURE 3:**
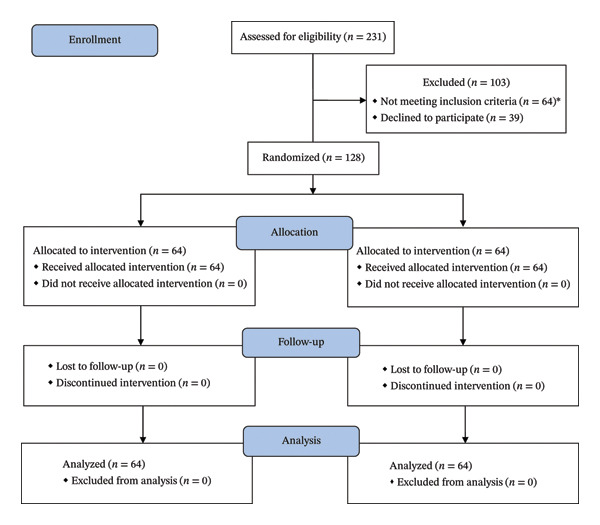
CONSORT 2010 flow diagram of patient enrollment. ^∗^Note: Not meeting inclusion criteria (*n* = 64), including neonatal comorbidity (*n* = 49), maternal comorbidity (*n* = 8), and inability to communicate using Mandarin Chinese or Taiwanese (*n* = 7).

From February 1, 2023, to December 31, 2023, 128 eligible mothers who agreed to participate in the study signed written informed consent forms. Participants were randomly divided into two groups through computer‐generated assignment, with 64 in the interventional group and 64 in the control group. All 128 mothers completed the entire study and provided full outcome information (Figure 3). No missing data were observed in this study; therefore, no imputation method was applied. Moreover, outcome assessors who were blinded to patient group allocation collected 100% of the outcomes through in‐person surveys and questionnaires.

### 3.1. Demographic and Clinical Data

Demographic information of participants of the interventional and control groups is summarized in Table 1. Regarding demographic factors, there were no differences noted between the two groups in terms of age, parity status, marital status, or occupation as a full‐time housewife. There was, however, a statistically significant difference in the educational level (*p* = 0.008). Regarding clinical variables, differences in previous breastfeeding experience, having previously participated in breastfeeding workshops, breastfeeding methods during hospitalization, and dominant hand for breastfeeding were not significant. In addition, there were no differences noted between the two groups in neonatal demographic data of birth height and weight and urinary frequency. However, there was a statistically significant difference in serum bilirubin levels on the day of discharge between the two infant groups (*p* = 0.03).

**TABLE 1:** Basic characteristics of the participants.

Demographic and clinical features	Intervention group (*N* = 64)	Control group (*N* = 64)	*p* value
Mothers			
Age (years)^a^	33.08 ± 4.12	32.91 ± 4.29	0.818
Parity status^b^			0.795
Primiparous	37 (57.8)	36 (56.3)	
Second parity	19 (29.7)	23 (35.9)	
Third parity	8 (12.5)	5 (7.8)	
Educational level^b^			0.008^∗^
Junior high school	3 (4.7)	0 (0)	
Senior high school	3 (4.7)	2 (3.1)	
University	50 (78.1)	44 (68.8)	
Master’s degree	8 (12.5)	18 (28.1)	
Marital status^b^			1.000
Married	64 (100)	64 (100)	
Single	0 (0)	0 (0)	
Occupation (full‐time housewife)^b^			0.806
Yes	55 (85.9)	54 (84.4)	
No	9 (14.1)	10 (15.6)	
Previous breastfeeding experience^b^			0.378
No	38 (59.4)	33 (51.6)	
Yes	26 (40.6)	31 (48.4)	
Experience participating in breastfeeding workshops^b^			0.575
No	58 (90.6)	56 (87.5)	
Yes	6 (9.4)	8 (12.5)	
Infant feeding method during hospitalization^b^			0.249
Exclusive breastfeeding	22 (34.4)	16 (25.0)	
Breastfeeding mixed with formula	42 (65.6)	48 (75.0)	
Dominant hand^b^			0.478
Left hand	6 (9.4)	4 (6.3)	
Right hand	46 (71.9)	53 (82.8)	
Both hands	11 (17.2)	7 (10.9)	
Neonatal			
Gestational age (weeks)^a^	38.53 ± 0.98	38.75 ± 1.18	0.26
Birth height (cm)^a^	49.92 ± 2.04	49.93 ± 1.76	0.98
Weight (gm)^a^			
Birth weigh	3046.66 ± 400.58	3082.03 ± 366.89	0.60
Second day	3011.00 ± 423.60 (*n* = 45)	3014.63 ± 359.81 (*n* = 40)	0.97
Third day	3030.63 ± 416.28 (*n* = 8)	3154.38 ± 488.32 (*n* = 8)	0.59
Day of discharge from hospital	2876.79 ± 507.83 (*n* = 63)	2949.69 ± 362.97 (*n* = 64)	0.35
Urinary frequency (times/day)^a^			
First day	2.05 ± 2.03	2.09 ± 2.03	0.90
Second day	4.86 ± 2.68	5.16 ± 2.41	0.51
Third day	5.17 ± 3.41 (*n* = 53)	5.20 ± 2.61 (*n* = 59)	0.95
Serum bilirubin level on the day of discharge (mg/dL)^a^	10.80 ± 2.59	9.85 ± 2.39	0.03^∗^

^a^Mean ± standard deviation.

^b^Count (%).

^∗^ *p* value < 0.05.

### 3.2. BPDS

The interventional group showed significantly higher comfort levels in all body parts during breastfeeding, with an average score of 3.52 ± 0.13 points, compared to the control group, which had an average score of 1.70 ± 0.33 points. The difference in breastfeeding comfort levels between the two groups was statistically significant (all *p* < 0.0001; Table 2).

**TABLE 2:** The Body Part Discomfort Scale (BPDS).

Body part	Interventional group (*N* = 64)	Control group (*N* = 64)	*p* value (*t*‐test)
Neck	3.21 ± 0.70	1.39 ± 0.49	< 0.0001^∗^
Left shoulder	3.40 ± 0.60	1.37 ± 0.51	< 0.0001^∗^
Right shoulder	3.42 ± 0.61	1.37 ± 0.51	< 0.0001^∗^
Left lower arms	3.53 ± 0.50	1.43 ± 0.53	< 0.0001^∗^
Right lower arms	3.53 ± 0.50	1.42 ± 0.52	< 0.0001^∗^
Mid back	3.59 ± 0.52	1.90 ± 0.49	< 0.0001^∗^
Lower back	3.60 ± 0.58	1.87 ± 0.52	< 0.0001^∗^
Buttocks	3.60 ± 0.55	1.89 ± 0.50	< 0.0001^∗^
Thighs	3.68 ± 0.46	2.17 ± 0.48	< 0.0001^∗^
Legs	3.62 ± 0.54	2.17 ± 0.48	< 0.0001^∗^
Average	3.52 ± 0.13	1.70 ± 0.33	< 0.0001^∗^

*Note:* Likert scale is scaled from 1 to 4, with 1 = *extremely uncomfortable* and 4 = *very comfortable*.

^∗^ *p* < 0.0001.

### 3.3. The BSES‐SF

In this study, the total score of the interventional group was 43.23 ± 10.78 points, while the total score of the control group was 42.52 ± 11.28 points. The difference between the two groups was not statistically significant (*p* = 0.71), indicating no difference in breastfeeding self‐efficacy between the two groups (Table 3).

**TABLE 3:** The Breastfeeding Self‐Efficacy Scale–Short Form (BSES‐SF).

Item	Intervention group (*N* = 64)	Control group (*N* = 64)	*p* value (*t*‐test)
I can always make sure that my baby gets enough breastmilk.	2.71 ± 1.07	2.65 ± 0.97	0.73
I can always successfully cope with breastfeeding, just like other challenging activities.	2.87 ± 1.04	2.87 ± 0.96	n.s.
I can always breastfeed my baby instead of using other commercial formulas as a substitute.	2.40 ± 1.10	2.25 ± 1.08	0.42
I can always make sure my baby is latched onto my breast completely during the whole feeding.	3.15 ± 1.07	3.42 ± 1.17	0.18
I can always deal with the breastfeeding situation to my satisfaction.	2.87 ± 0.95	2.81 ± 1.02	0.72
I can always breastfeed even when my baby is crying.	3.14 ± 1.06	3.09 ± 1.17	0.81
I can always keep the desire to breastfeed.	3.29 ± 1.04	3.12 ± 1.18	0.38
I can always breastfeed comfortably while my family is present.	3.45 ± 1.00	3.29 ± 1.19	0.42
I can always be satisfied with my breastfeeding experience.	3.07 ± 0.96	3.07 ± 1.13	n.s.
I can always deal with the fact that it is time‐consuming to breastfeed.	3.46 ± 0.90	3.50 ± 1.06	0.85
I can always finish breastfeeding on one side of the breast before switching to the other side.	3.53 ± 1.08	3.48 ± 1.20	0.81
I can always continue to breastfeed my baby for every feeding.	3.23 ± 0.98	3.03 ± 1.20	0.26
I can always work to keep up with my baby’s needs to breastfeed.	3.23 ± 1.15	3.26 ± 1.11	0.87
I can always tell when my baby has finished breastfeeding.	2.76 ± 1.0	2.62 ± 1.03	0.44
Average	3.10 ± 0.34	3.04 ± 0.36	0.70
Total	43.23 ± 10.78	42.52 ± 11.28	0.71

*Note:* The Breastfeeding Self‐Efficacy Scale–Short Form is scored from 1 to 5 and was completed on the day of discharge; 1 = *not at all confident* and 5 = *always confident*.

### 3.4. BAS

The average total score for the interventional group was 3.58 ± 0.04 points, while the control group had an average score of 1.60 ± 0.15 points for each question. There was a significant difference in BAS 4 questions between the two groups (all *p* < 0.0001), suggesting that mothers in the intervention group experienced greater awareness and reassurance during breastfeeding (Table 4).

**TABLE 4:** Breastfeeding Awareness Scale (BAS).

Item	Intervention group (*N* = 64)	Control group (*N* = 64)	*p* value (*t*‐test)
I feel comfortable falling asleep while holding my baby while breastfeeding.	3.56 ± 0.53	1.51 ± 0.59	< 0.0001^∗^
I feel reassured about maintaining the correct nursing position during breastfeeding.	3.54 ± 0.56	1.73 ± 0.47	< 0.0001^∗^
I feel reassured about the baby’s safety, such as falling, during breastfeeding.	3.57 ± 0.61	1.42 ± 0.49	< 0.0001^∗^
I feel relaxed and at ease during breastfeeding.	3.65 ± 0.54	1.73 ± 0.44	< 0.0001^∗^
Average	3.58 ± 0.04	1.60 ± 0.15	< 0.0001^∗^

*Note:* Likert scale is scaled from 1 to 4 with 1 = *extremely worried* and 4 = *very secure*.

^∗^ *p* < 0.0001.

### 3.5. B‐R‐E‐A‐S‐T‐Feed Observation Form

Results indicated that the interventional and control groups both scored higher on emotional bonding during the initial assessment (T0) (2.60 ± 0.49 vs. 2.17 ± 0.60; *p* < 0.0001) and on the day of discharge (T1) (2.87 ± 0.33 vs. 2.48 ± 0.56; *p* < 0.0001). In particular, “Much touching by mother” received higher scores on the day of discharge (0.95 ± 0.21 vs 0.54 ± 0.50; *p* < 0.0001) compared to their initial assessment (0.76 ± 0.42 vs 0.40 ± 0.49; *p* < 0.0001), suggesting an enhanced breastfeeding experience (Table 5).

**TABLE 5:** B‐R‐E‐A‐S‐T‐Feed Observation Form.

Signs that breastfeeding is going well (scored 1 point)	Initial assessment (T0)			Day of discharge (T1)		
	Intervention group (*N* = 64)	Control group (*N* = 64)	Between group *p* value	Intervention group (*N* = 64)	Control group (*N* = 64)	Between group *p* value
Body position	4.84 ± 0.40	4.79 ± 0.50	0.56	4.98 ± 0.12	4.95 ± 0.21	0.31
Mother relaxed and comfortable	0.90 ± 0.29	0.84 ± 0.36	0.28	0.98 ± 0.12	0.98 ± 0.12	> 0.99
Baby’s body close, facing breast	0.98 ± 0.12	0.98 ± 0.12	> 0.99	1.00 ± 0.00	1.00 ± 0.00	1.00
Baby’s head and body straight	1.00 ± 0.00	0.98 ± 0.12	0.31	1.00 ± 0.00	0.96 ± 0.17	0.15
Baby’s chin touching breast	0.98 ± 0.12	0.98 ± 0.12	> 0.99	1.00 ± 0.00	1.00 ± 0.00	1.00
Baby’s bottom supported	0.96 ± 0.17	1.00 ± 0.00	0.15	1.00 ± 0.00	1.00 ± 0.00	1.00
Responses	4.67 ± 1.23	4.81 ± 1.15	0.50	5.56 ± 0.70	5.50 ± 0.75	0.63
Baby reaches for breast if hungry	0.87 ± 0.33	0.93 ± 0.24	0.22	0.96 ± 0.17	0.98 ± 0.12	0.56
Baby roots for breast	0.92 ± 0.27	0.93 ± 0.24	0.73	0.98 ± 0.12	0.98 ± 0.12	> 0.99
Baby explores breast with tongue	0.87 ± 0.33	0.90 ± 0.29	0.57	1.00 ± 0.00	0.98 ± 0.12	0.32
Baby calm and alert at breast	0.65 ± 0.47	0.82 ± 0.38	0.02	0.90 ± 0.29	0.95 ± 0.21	0.30
Baby stays attached to the breast	0.87 ± 0.33	0.82 ± 0.38	0.45	1.00 ± 0.00	0.98 ± 0.12	0.31
Signs of milk ejection, leaking, afterpain	0.46 ± 0.50	0.37 ± 0.48	0.28	0.70 ± 0.46	0.60 ± 0.49	0.26
Emotional bonding	2.60 ± 0.49	2.17 ± 0.60	< 0.0001^∗^	2.87 ± 0.33	2.48 ± 0.56	< 0.0001^∗^
Secure, confident hold	0.87 ± 0.33	0.78 ± 0.42	0.16	0.95 ± 0.21	0.95 ± 0.21	> 0.99
Face‐to‐face attention from mother	0.96 ± 0.17	0.98 ± 0.13	0.56	0.96 ± 0.17	0.98 ± 0.12	0.56
Much touching by mother	0.76 ± 0.42	0.40 ± 0.49	< 0.0001^∗^	0.95 ± 0.21	0.54 ± 0.50	< 0.0001^∗^
Anatomy	3.98 ± 0.12	4.00 ± 0.00	0.31	3.95 ± 0.21	3.92 ± 0.27	0.46
Breasts soft after feeding	1.00 ± 0.00	1.00 ± 0.00	1.00	1.00 ± 0.00	1.00 ± 0.00	1.00
Nipples stand out, protractile	0.98 ± 0.12	1.00 ± 0.00	0.31	1.00 ± 0.00	0.98 ± 0.12	0.31
Skin appears healthy	1.00 ± 0.00	1.00 ± 0.00	1.00	0.95 ± 0.21	0.93 ± 0.24	0.70
Breast looks round during feeding	1.00 ± 0.00	1.00 ± 0.00	1.00	1.00 ± 0.00	1.00 ± 0.00	1.00
Suckling	6.40 ± 1.37	6.65 ± 1.05	0.25	6.96 ± 0.17	7.00 ± 0.00	0.15
Mouth wide open	0.87 ± 0.33	0.93 ± 0.24	0.22	1.00 ± 0.00	1.00 ± 0.00	1.00
Lower lip turned outward	0.89 ± 0.31	0.93 ± 0.24	0.34	1.00 ± 0.00	1.00 ± 0.00	1.00
Tongue cupped around breast	1.00 ± 0.00	1.00 ± 0.00	1.00	1.00 ± 0.00	1.00 ± 0.00	1.00
Cheeks round	0.98 ± 0.12	0.95 ± 0.21	0.31	1.00 ± 0.00	1.00 ± 0.00	1.00
More areola above baby’s mouth	0.88 ± 0.33	0.95 ± 0.21	0.11	0.98 ± 0.13	1.00 ± 0.00	0.31
Slow deep sucks, bursts with pauses	0.85 ± 0.39	0.94 ± 0.24	0.17	1.00 ± 0.00	1.00 ± 0.00	1.00
Can see or hear swallowing	0.92 ± 0.32	0.93 ± 0.24	0.75	0.98 ± 0.12	1.00 ± 0.00	0.31
Time spent suckling						
Baby releases breast	0.96 ± 0.17	1.00 ± 0.00	0.15	1.00 ± 0.00	1.00 ± 0.00	1.00
Baby suckled for ___ minutes	21.36 ± 12.73	23.53 ± 13.46	0.35	29.22 ± 13.6	27.81 ± 10.11	0.50

^∗^ *p* < 0.001.

### 3.6. Regression Analysis on the BPDS

Mothers’ educational levels and newborns’ serum bilirubin levels on the day of discharge demonstrated significant differences (*p* = 0.008 and *p* = 0.03, respectively) between the two groups (Table 1). A regression analysis was performed, incorporating educational level, study group, and newborn serum bilirubin levels to assess the BPDS. Results for educational level showed no significant differences between the university level (*p* = 0.77) and the master’s degree level (*p* = 0.59), as well as for the bilirubin level (*p* = 0.18). When the groups were compared, the interventional group demonstrated a significantly higher breastfeeding comfort level of 18.13 points higher than the control group (*p* < 0.001; Table 6).

**TABLE 6:** Regression analysis on the Body Part Discomfort Scale (BPDS).

Variable	B	SE	β	T	*p*
Education level (Reference group: junior and senior high school)					
University	−0.41	1.40	−0.02	−0.29	0.77
Master’s degree	−0.84	1.56	−0.03	−0.54	0.59
Group (Reference group: control group)					
Interventional group	18.13	0.69	0.92	26.46^∗^	< 0.001^∗^
Newborn serum bilirubin levels on the day of discharge	−0.18	0.14	−0.05	−1.34	0.18

*Note: F* = 187.63, *p* < 0.001; *R* ^2^ = 0.86, adjusted *R* ^2^ = 0.86.

Abbreviation: SE, standard error.

^∗^ *p* < 0.001.

## 4. Discussion

In this study, we demonstrated several key advantages of the nurse‐designed innovative Safe Breastfeeding Pillow. The pillow can be wrapped around the infant to maintain a stable feeding posture, thereby reducing the risk of accidents related to maternal fatigue during breastfeeding. Utilizing the innovative Safe Breastfeeding Pillow can effectively enhance maternal breastfeeding safety and comfort and optimize the overall breastfeeding outcome compared to a traditional breastfeeding pillow. The current results using Likert scale measures of breastfeeding comfort level and awareness indicated that mothers in the interventional group experienced greater comfort and reassurance, while the BPDS revealed significantly higher comfort levels in all body parts during breastfeeding. There was a lack of significant difference in breastfeeding self‐efficacy between groups. This finding may suggest that breastfeeding self‐efficacy, as a multidimensional and relatively stable psychological construct, is less susceptible to short‐term changes induced by a single supportive device, highlighting the need for sustained and multifaceted interventions to enhance maternal confidence. According to Tanrıverdi et al. [10], breastfeeding self‐efficacy is influenced by multiple interventions, including education, counseling, and peer support. Therefore, changes in breastfeeding self‐efficacy often require sustained experiences or educational interventions rather than short‐term exposure to a single device or tool. Additionally, the exceptionally high explanatory power (*R* ^2^ = 0.86) observed in the regression model may primarily reflect the pronounced difference in BPDS scores between the intervention and control groups rather than a complex model structure. This strong effect size could indicate potential overfitting, underscoring the importance of validating these findings in larger and more diverse samples in future studies. Moreover, incorporating the Safe Breastfeeding Pillow into routine care may enhance feeding safety, maternal comfort, and breastfeeding outcomes, highlighting its clinical relevance for supporting mothers and infants in the postpartum period.

In the past, many mothers discontinued breastfeeding due to challenges in maintaining a comfortable and proper breastfeeding posture. This often resulted in issues such as cracked nipples, insufficient milk supply, pain, and exhaustion when using a traditional breastfeeding pillow [7]. Additionally, traditional breastfeeding pillows that need continuous hand support from the mother are essential during nighttime feedings when there is a risk of unintentional incidents if the mother falls asleep which has raised concerns about maternal and infant safety. To address these issues, our team designed a first‐generation, innovative Safe Breastfeeding Pillow. In February 2019, this innovative breastfeeding pillow featuring fixed supplementary pillows was tested by postpartum mothers in the nursery. User feedback, particularly regarding insufficient support for smaller babies weighing under 2500 g, prompted us to conduct a comprehensive review and implement adjustments. In April 2019, we redesigned the first‐generation breastfeeding pillow, incorporating a detachable small blanket secured with Velcro. This blanket can mimic the mother’s hands by wrapping around the baby to help the baby achieve a secure feeding position, ensuring safety and comfort during nursing while preventing the mother from becoming tired. It can also function as a warm blanket during nighttime feedings or when the baby’s temperature is lower than desired. We made innovative improvements to produce a user‐friendly product that requires no specialized training, leading to increased breastfeeding safety and medical satisfaction, and elevating the quality of care for mothers and infants.

Breastfeeding is characterized by important psychological consequences for neonates and mothers [14–17]. In addition to providing nutrients, breast milk is rich in microbiota and nonimmune and immune components to ensure the infant’s protection against numerous diseases and support maturation of the infant’s developing immune system [18, 19]. Ensuring successful breastfeeding involves not only providing infants with an adequate milk supply but also ensuring the comfort and awareness of mothers. Discomfort and worries experienced by participants in the control group during breastfeeding arose from insufficient arm support, leading to prolonged contraction of shoulder muscles and resulting in muscle stiffness. Postpartum mothers’ condition can exacerbate fatigue and discomfort during breastfeeding [20]. Breastfeeding pillows contribute to maternal comfort and relaxation during breastfeeding by effectively supporting the baby’s weight and preventing fatigue in the mother’s arms, shoulders, and neck [21]. By alleviating muscle strain and ensuring a sufficient oxygen supply, breastfeeding pillows help maintain the mother’s physical well‐being during breastfeeding sessions. Measuring discomfort across various body parts using the BPDS revealed that the interventional group reported significantly higher comfort levels in all body parts during breastfeeding compared to the control group, consistent with our study findings. Other research and related studies have also shown a 5.73% reduction in maternal fatigue postbreastfeeding when utilizing breastfeeding pillows [7].

Breastfeeding, endorsed by both the World Health Organization (WHO) and the UNICEF, stands as the gold standard for infant nutrition. The WHO and UNICEF advocate exclusive breastfeeding in the initial 6 months after birth, followed by continued breastfeeding alongside complementary foods for at least 2 years [9]. However, maintaining breastfeeding upon a mother’s return to work presents challenges. To address this, family members can play a pivotal role by utilizing the innovative Safe Breastfeeding Pillow to assist with feeding. This versatile tool supports both breastfeeding and bottle feeding as needed, ensuring the baby receives essential nutrition even when the mother is absent. Additionally, the natural bioactive components found in human milk enhance immunity in the infant during the first years of life [22]. Moreover, the use of the innovative Safe Breastfeeding Pillow enhances comfort and awareness during feeding sessions, fostering a nurturing environment for both mother and baby. This collaborative approach to feeding facilitates the mother’s transition back to work while prioritizing the baby’s nutritional requirements and strengthening familial bonds.

To support effective implementation in real‐world settings, nurse leaders can take an active role in introducing the innovative Safe Breastfeeding Pillow and ensuring that staff receive hands‐on training in appropriate positioning, maternal comfort assessment, and breastfeeding support techniques. The integration and sustainability of the innovative Safe Breastfeeding Pillow in routine breastfeeding care may be supported by the standard of procedures, ongoing supervision, and continuous evaluation of maternal comfort, breastfeeding experiences, and feedback from both nursing staff and mothers. Potential barriers to implementation may include limited resources, staff unfamiliarity with the new device, resistance to changes in routine care, and variation in mothers’ individual needs and breastfeeding experiences. To address these challenges, nurse leaders may adopt phased implementation, encourage frontline staff engagement, provide continuous support and training, and use quality improvement processes to monitor outcomes and guide practice refinement.

## 5. Limitations

This study was conducted during the Coronavirus Disease 2019 (COVID‐19) pandemic period in a nursery room with an adequate number of clinicians and paramedical staff support for the breastfeeding‐aid explanations; moreover, the sample size was sufficiently large to afford statistically powerful results. However, there are several limitations to consider. First of all, the healthcare providers who distributed the breastfeeding pillows were not blinded, which may have introduced potential bias, efforts were made to minimize this influence by ensuring that outcome assessors and participants remained blinded to group allocation. Second, due to participating mothers’ data were collected in very specific nursery settings, the outcomes might not be generalizable to different environment settings. The potential for implementing the pillow in other community settings, particularly home‐birth environments, warrants further investigation. Nevertheless, the innovative Safe Breastfeeding Pillow with adequate instructions on how to use it can be utilized in outside settings. Mothers who underwent a cesarean section or had emergency conditions were excluded from this study. Third, infants with a birth weight of < 2000 g were not included, and there were no participants with birth weights of 2000–2600 g, despite meeting the selection criteria. Furthermore, as the study focused on newborns within the early postpartum period, the application of Safe Breastfeeding Pillow to infants older than 28 days or those weighing more than 6 kg may need further evaluation. Fourth, the BAS and BPDS scores exhibited a potential ceiling effect, suggesting limited discriminative power of these self‐developed scales. Future studies are encouraged to employ or develop more comprehensive and psychometrically validated instruments to enhance measurement precision. Last, the duration of breastfeeding evaluated in the study was constrained by the postpartum hospital stay duration, which can vary across healthcare facilities.

## 6. Conclusions

In conclusion, this study investigated the effects of an innovative Safe Breastfeeding Pillow compared to a traditional one among postpartum mothers during breastfeeding. While both groups showed similar breastfeeding efficacies, mothers using the Safe Breastfeeding Pillow reported notably higher comfort and reassurance levels, with significantly higher comfort levels in all body parts during breastfeeding. As a result, we recommend adopting the innovative Safety Breastfeeding Pillow by mothers and caregivers to ensure safe and comfortable nursing experiences.

## Funding

This work was supported by a research grant from Shuang Ho Hospital, Taipei Medical University (grant no. 111TMU‐SHH‐34).

## Disclosure

The sponsoring organization was not involved in the study design, data analysis, or interpretation.

## Conflicts of Interest

Head Nurse Ju‐Fen Chou received research funding from Shuang Ho Hospital, Taipei Medical University (grant no. 111TMU‐SHH‐34). The other authors declare no conflicts of interest.

## Data Availability

The data that support the findings of this study are available from the corresponding author upon reasonable request.
